# Activation of heat shock response to treat obese subjects with type 2 diabetes: a prospective, frequency-escalating, randomized, open-label, triple-arm trial

**DOI:** 10.1038/srep35690

**Published:** 2016-10-19

**Authors:** Tatsuya Kondo, Rieko Goto, Kaoru Ono, Sayaka Kitano, Mary Ann Suico, Miki Sato, Motoyuki Igata, Junji Kawashima, Hiroyuki Motoshima, Takeshi Matsumura, Hirofumi Kai, Eiichi Araki

**Affiliations:** 1 Department of Metabolic Medicine, Faculty of Life Sciences, Kumamoto University, 1-1-1 Honjo, Chuo-Ward, Kumamoto 860-8556, Japan; 2 Department of Molecular Medicine, Faculty of Life Sciences, Kumamoto University, 5-1 Oe, Chuo-Ward, Kumamoto 862-0973, Japan

## Abstract

Activation of heat shock response (HSR) improves accumulated visceral adiposity and metabolic abnormalities in type 2 diabetes. To identify the optimal intervention strategy of the activation of the HSR provided by mild electrical stimulation (MES) with heat shock (HS) in type 2 diabetes. This study was a prospective, frequency-escalating, randomized, open-label, triple-arm trial in Japan. A total of 60 obese type 2 diabetes patients were randomized into three groups receiving two, four, or seven treatments per week for 12 weeks. No adverse events were identified. MES + HS treatment (when all three groups were combined), significantly improved visceral adiposity, glycemic control, insulin resistance, systemic inflammation, renal function, hepatic steatosis and lipid profile compared to baseline. The reduction in HbA1c was significantly greater among those treated four times per week (−0.36%) or seven times per week (−0.65%) than among those treated two times per week (−0.10%). The relative HbA1c levels in seven times per week group was significantly decreased when adjusted by two times per week group (−0.55%. p = 0.001). This research provides the positive impact of MES + HS to treat obese patients with type 2 diabetes mellitus.

The pandemic of type 2 diabetes mellitus has negative health impacts worldwide, and is associated with the significant expansion of obesity, which is particularly characterized by increased visceral adiposity with chronic systemic inflammation. As anti-diabetic pharmacotherapy usually becomes insufficient to control glucose metabolism against the progression of insulin resistance and β-cell failure, many patients require additional interventions such as multiple oral drugs and/or injection therapies^1^, which do not reverse the fundamental pathophysiology of diabetes.

One underlying mechanism supporting the development of type 2 diabetes among obese individuals and the worsening of glucose control is the attenuation of the heat shock response (HSR), which is closely associated with heat shock protein (HSP) 72 expression. HSP72 acts as an anti-inflammatory, anti-apoptotic, and cell-protective molecule^2^ and the regulation of HSP72 expression is tightly related to insulin signaling^3^. Chronic systemic inflammation caused by visceral adiposity promotes insulin resistance. The impaired insulin signaling in turn reduces the cytoplasmic abundance of HSP72, resulting in damage to the pancreatic β-cells and further attenuation of insulin signaling^4^.

Adding to HSP72 levels with various modalities, including mild electrical stimulation (MES) plus heat shock (HS), improved visceral adiposity, glucose homeostasis, and chronic systemic inflammation^5^,^6^,^7^,^8^,^9^. We have conducted a preliminary intervention study using MES + HS for subjects with metabolic syndrome or obese type 2 diabetes, and identified that this method activates the HSR and improved visceral adipose mass, glucose homeostasis, and inflammatory surrogate markers including tumor necrosis factor (TNF)-α and C-reactive protein (CRP)^6^, which are quite similar to the effects observed in diabetic animal models treated with MES + HS^7^,^9^.

In this manuscript, we investigate the optimal clinical applications of this MES + HS treatment for obese subjects with type 2 diabetes to bring this intervention into clinical settings.

## Results

### Baseline characteristics of the obese type 2 diabetes patients

Demographic characteristics, including concomitant medications, adiposity, blood pressure, glucose control, systemic inflammation, renal function, and hepatic steatosis, were not significantly different among the three treatment groups (Table 1). No serious adverse effects or hypoglycemia over the 12 weeks of intervention were found.

### Primary endpoints: the amount of visceral adiposity, glucose control, and insulin resistance

#### Effects of MES + HS over time compared with the baseline

**Figure 1:**
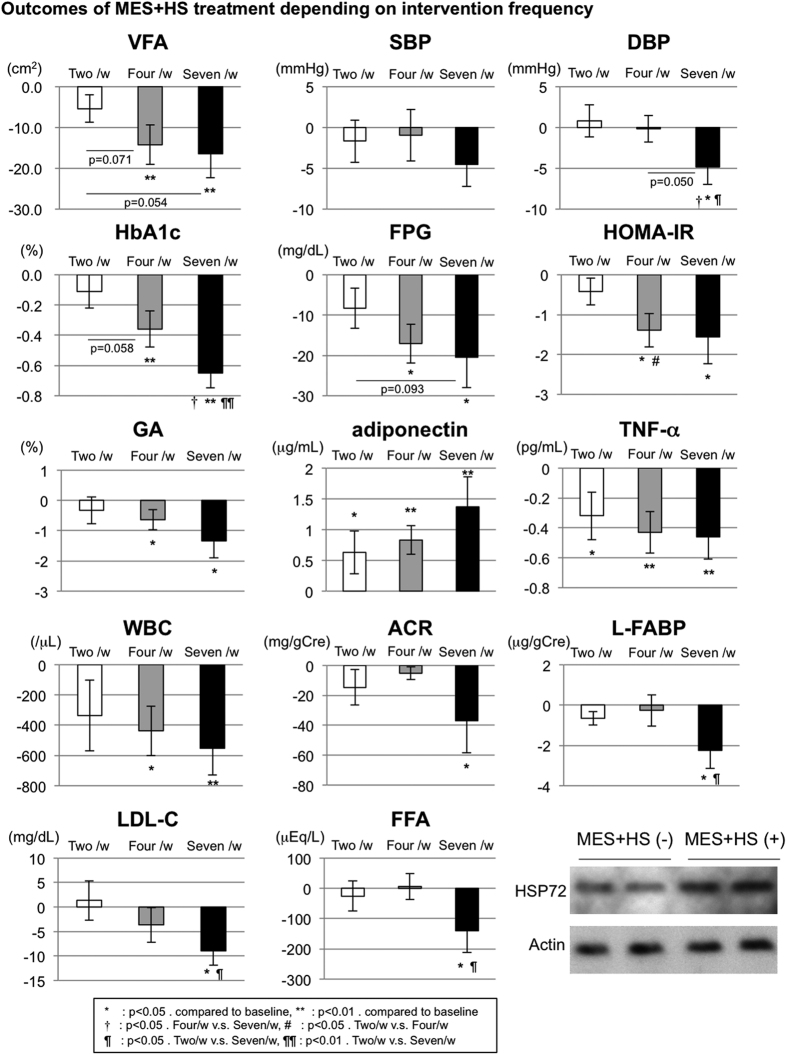
Outcomes of MES + HS treatment depending on intervention frequency. The absolute changes (average with standard error of the mean) of the markers such as VFA (visceral fat area), SBP (systolic blood pressure), DBP (Diastolic blood pressure), HbA1c (glycated hemoglobin), FPG (fasting plasma glucose), HOMA-IR (homeostasis model assessment as an index of insulin resistance), GA (glycated albumin), adiponectin, TNF-α (tumor necrosis factor- α), WBC (white blood cell count), ACR (albumin creatinine ratio), L-FABP (liver-type fatty acid binding protein), LDL-C (low density lipoprotein cholesterol) and FFA (free fatty acid) on the MES + HS treatment frequency (two, four or seven/w) from each baseline were indicated. The numbers in group: two/w: n = 22, four/w: n = 19, seven/w: n = 19. *p < 0.05. compared to baseline. **p < 0.01. compared to baseline. ^†^p < 0.05. Four/w v.s. Seven/w. ^#^p < 0.05. Two/w v.s. Four/w. ^¶^p < 0.05. Two/w v.s. Seven/w. ^¶¶^p < 0.01. Two/w v.s. Seven/w. Lower right panels indicate HSP72 protein expression in monocytes isolated from pre (MES + HS (−)) and after (MES + HS (+)) the treatment of MES + HS (seven times per week).

From randomly selected 6 subjects, HSP72 expression after the treatment was increased approximately 1.5 times in isolated monocytes compared to before the treatment (Fig. 1. lower right panels) Table 2. This indicates that the activation of HSR was similar levels in our previous study^6^.

##### Adiposity

The visceral fat area (VFA), measured by computed tomography scan, decreased by 11.69 cm^2^ (from 166.12 ± 7.75 to 154.44 ± 6.76 cm^2^; p < 0.001) following MES + HS treatment compared with the baseline value, while the subcutaneous fat area (SFA) was not changed (from 207.11 ± 11.95 to 204.04 ± 12.14 cm^2^; −3.07 cm^2^; p = 0.205). As a result, the total fat area decreased significantly by 14.75 cm^2^ following MES + HS treatment. Body mass index (BMI: from 29.14 ± 0.56 to 28.88 ± 0.58 kg/m^2^; −0.25 kg/m^2^; p = 0.001) and waist circumference (Wc: from 100.10 ± 1.33 to 97.86 ± 1.23; −2.24 cm. p < 0.001) both also decreased.

##### Glucose control and insulin resistance

Fasting plasma glucose (FPG) reduced from 159.38 ± 5.30 to 144.45 ± 4.21 mg/dL (−14.93 mg/dL, p < 0.001). Fasting immune-reactive insulin also decreased, from 9.77 ± 0.74 to 7.91 ± 0.59 μIU/mL (−1.86 μIU/mL, p < 0.001). Hence, HOMA-IR improved from 4.08 ± 0.42 to 2.99 ± 0.29 (−1.09, p < 0.001). HbA1c declined from 7.64 ± 0.08% to 7.28 ± 0.08% (−0.36 ± 0.07%, p < 0.001) and glycated albumin (GA) also dropped from 19.15 ± 0.41 to 18.40 ± 0.39% (−0.75%, p = 0.004). As a result, the clinical target of HbA1c less than 7.0% was achieved by 38.3% (*n* = 23) of participants after MES + HS treatment. Adiponectin increased from 7.54 ± 0.47 to 8.47 ± 0.54 μg/mL (+0.93 μg/mL, p < 0.001). Furthermore, multiple regression analysis indicated that the decrease of VFA was significantly correlated with the amount of VFA before the treatment (r = 0.565, p < 0.001) and the reduction of HbA1c was significantly correlated with baseline HbA1c (r = 0.485, p = 0.002).

#### Effects of MES + HS depending on treatment frequency

MES + HS treatment does not have appropriate placebo control because this apparatus transmits heat and electric stimulation simultaneously, and these different frequencies of intervention may be necessary to identify the exact clinical effects of MES + HS (Fig. 1 and Table 3).

##### Adiposity

VFA was compared among two, four, and seven treatments per week, and the amplitude of reduction was −5.37, −14.24, and −16.45 cm^2^, respectively (Fig. 1). The trend of VFA decreases in seven treatments per week group was observed compared with that in two treatments per week group (p = 0.054). This trend was also observed in four treatments per week group compared with the two treatments per week (p = 0.071). There were no significant differences between the four and seven treatments per week groups. The changes in Wc showed similar trends, but SFA did not.

##### Glucose control and insulin resistance

FPG indicated a trend toward decreasing in the seven treatments per week group compared with that in the two treatments per week group (−8.32 vs. −20.47 mg/dL, p = 0.093. Fig. 1). HOMA-IR showed a significant decline in the four treatments per week group compared with that in the two treatments per week group (p = 0.043). Reductions in HbA1c following MES + HS in two, four, and seven treatments per week were −0.10 ± 0.11%, −0.36% ± 0.12 and −0.65% ± 0.10, respectively (Fig. 1). The decrease of HbA1c in the seven treatments per week group was significantly greater than that in the two or four treatments per week group (2 vs. 7, *p* = 0.001; 4 vs. 7, p = 0.036). GA showed a similar trend in reduction, but did not reach a significant difference. Adiponectin levels were increased over time in all groups, but were not different among the three groups.

#### Effects of MES + HS in sex differences

##### Adiposity

VFA significantly decreased from 177.28 ± 10.25 to 161.72 ± 8.87 cm^2^ (−15.55 cm^2^, p < 0.001, *n* = 48) in male but not female participants (−2.66 cm^2^, p = 0.262, *n* = 22). The amplitude of VFA reduction was significantly larger in male than in female participants (p = 0.017). SFA showed a trend toward reduction in female participants (−10.59 cm^2^, p = 0.068), but not in male participants (+0.16 cm^2^, p = 0.485). BMI (−0.30 kg/m^2^, p = 0.003) and Wc (−2.67 cm, p < 0.001) were both decreased only in men, but the sex differences were not obvious (Table 4).

##### Glucose control and insulin resistance

FPG (from 167.50 ± 6.86 to 145.31 ± 5.27 mg/dL, −22.19 mg/dL, p < 0.001) and HOMA-IR (from 4.23 ± 0.56 to 2.92 ± 0.37, −1.31, p < 0.001) both significantly improved only in males. The sex difference in FPG was significant (p < 0.001). HbA1c showed a minor trend toward reduction in female participants (Δ = −0.17 ± 0.15%, p = 0.140) and a significant decrease in male participants (Δ = −0.44 ± 0.07%, p < 0.001). The difference in the reduction of HbA1c was obvious (p = 0.033). GA indicated a similar change of +0.12% (p = 0.384) in female and −1.12% (p < 0.001) in male participants, and the difference was significant (p = 0.017). However, the level of adiponectin increased both in female (+1.07 μg/mL; p = 0.011) and male participants (+0.87 μg/mL; p < 0.001).

### Secondary outcomes: blood pressure, systemic inflammation, renal function, hepatic steatosis, and lipids

#### Effects of MES + HS over time compared with baseline

##### Systemic inflammation

Tumor necrosis factor (TNF)-α levels in serum decreased from 1.64 ± 0.11 to 1.24 ± 0.08 pg/mL (−0.40 pg/mL; p < 0.001). Interleukin (IL)-6 was not changed significantly (from 3.17 ± 0.29 to 2.78 ± 0.31 pg/mL, −0.44 pg/mL; p = 0.105). C-reactive protein (CRP) decreased from 1968.77 ± 442.90 to 1305.17 ± 246.04 ng/mL (−663.60 ng/mL; p = 0.008) and the counts of white blood cells (WBC) also decreased from 6751.67 ± 204.86 to 6315.00 ± 183.01/μL (−436.67/μL; p < 0.001) (Table 2).

##### Renal function

The estimated glomerular filtration rate (eGFR) was elevated from 76.52 ± 2.23 to 79.48 ± 2.35 mL/min/1.73 m^2^ (+2.96 mL/min/1.73 m^2^; p < 0.001). Renal albumin excretion, estimated by urinary albumin creatinine ratio (ACR) was reduced from 75.30 ± 20.87 to 56.47 ± 15.82 mg/g Cre (−18.73 mg/g Cre; p = 0.015). Oxidative stress marker in renal tubules, evaluated by liver-type fatty acid-binding protein (L-FABP), was decreased from 5.42 ± 0.53 to 4.38 ± 0.49 μg/g Cre (−1.04 μg/g Cre; p = 0.007) as well.

##### Hepatic steatosis and lipids

The AST/ALT ratio increased from 0.86 ± 0.03 to 0.92 ± 0.04 (+0.06; p = 0.007). Uric acid (UA) slightly, but significantly, decreased from 5.58 ± 0.19 to 5.43 ± 0.18 mg/dL (−0.15 mg/dL; p = 0.042). Low-density lipoprotein cholesterol (LDL-C) showed a trend toward reduction (from 109.30 ± 3.54 to 105.80 ± 3.45 mg/dL, −3.50 mg/dL, p = 0.055). High-density lipoprotein cholesterol (HDL-C) and free fatty acid (FFA) were not changed by MES + HS treatment. Triglyceride (TG) levels were significantly reduced from 183.40 ± 18.75 to 153.38 ± 12.46 mg/dL (−30.02 mg/dL; p = 0.015).

#### Effects of MES + HS depending on frequency of treatment

##### Blood pressure

A decrease in diastolic blood pressure (DBP) but not systolic (SBP) was observed in the seven treatments per week compared with that in the two or four treatments per week group (p = 0.050 and 0.033, respectively. Figure 1) (Fig. 1 and Table 3).

##### Systemic inflammation

Although chronic inflammatory markers such as TNF-α, IL-6, CRP, and the WBC count decreased over time in all intervention groups, the differences among groups were not statistically significant (Fig. 1).

##### Renal function

eGFR was elevated and ACR and L-FABP were decreased in every intervention group, but a significant difference was observed only in L-FABP in the seven treatments per week group compared with that of two treatments per week group (p = 0.046 Fig. 1).

##### Hepatic steatosis and lipids

Almost all markers of hepatic steatosis (AST/ALT ratio), UA, and lipid profiles showed favorable trends of changes in all groups. Significant reductions in LDL-C were observed in the seven treatments per week compared with that in the two treatments per week group (p = 0.028. Fig. 1).

#### Effects of MES + HS in sex differences

##### Blood pressure

The decreases in SBP (−4.38 mmHg; p = 0.012) and DBP (−2.74 mmHg; p = 0.006) were observed only in male participants, and the sex differences were statistically significant (p = 0.030 and p = 0.029, respectively). Heart rates were also reduced only in male participants (−2.31 beats/min; p = 0.044) (Table 4).

##### Systemic inflammation

Although TNF-α decreased in both female (−0.36 pg/mL; p = 0.020) and male participants (−0.42 pg/mL; p < 0.001), IL-6 (−0.87 pg/mL; p < 0.001), CRP (−907.26 ng/mL; p = 0.001), and WBC (−478.57/μL; p < 0.001) were reduced only in males. The sex difference in reduction of IL-6 was significant (p = 0.008).

##### Renal function

eGFR (+2.33 mL/min/1.73 m^2^ in women, p = 0.034; +2.66 mL/min/1.73 m^2^ in men, p = 0.001) and L-FABP (−0.97 μg/g Cre in women, p = 0.025; −1.06 μg/g Cre in men, p = 0.032) were ameliorated in female as well as male participants. A sex difference was not observed.

##### Hepatic steatosis and lipids

Amelioration of the AST/ALT ratio (+0.07; p = 0.019), UA (−0.18 mg/dL; p = 0.043), HDL-C (+2.14 mg/dL; p = 0.037), TG (−22.07 mg/dL; p = 0.036), and free fatty acid (FFA: −70.40 μEq/L; p = 0.038) was observed only in male participants. Significant sex differences were observed in the increases of HDL-C (p = 0.011).

#### Sub-analysis with or without DPP-4 inhibitors

In most cases, the favorable changes in biophysical and biochemical parameters were not influenced by concomitant anti-diabetic medications. However, FPG was significantly reduced from 162.16 ± 6.96 to 142.29 ± 5.41 mg/dL (−19.87 mg/dL; p < 0.001) in subjects prescribed with DPP-4 inhibitors, but only a trend in those without DPP-4 inhibitors (−6.41 mg/dL; p = 0.082). The reduction in FPG was significantly greater in subjects with DPP-4 inhibitors than those in without DPP-4 inhibitors (p = 0.030). A significant difference was observed in the decrease of L-FABP (−1.56 μg/g Cre; p = 0.049 vs. without DPP-4 inhibitors) and in the increase of the AST/ALT ratio (+0.09; p = 0.031 vs. without DPP-4 inhibitors) in subjects with DPP-4 inhibitors (Supplementary Table 3).

## Discussion

For patients who are obese and have type 2 diabetes, a therapeutic strategy to modify the progression of the disease and complications beyond the control of glucose levels may provide additional long-term health benefits. The preferable metabolic effects of MES + HS treatment in obese patients with type 2 diabetes were regulated by the frequency of intervention. In particular, the effect on HbA1c levels was clearly intervention frequency dependent (−0.10%, −0.36%, and −0.65%, respectively). Another primary endpoint, VFA also showed a trend in reduction depending on the treatment frequency (−5.37 cm^2^, −14.24 cm^2^, and −16.45 cm^2^, respectively). Although MES + HS treatment cannot set a precise placebo control, these frequency-dependent favorable results indicate that MES + HS correctly functions to improve metabolic abnormalities in obese subjects with type 2 diabetes.

### Reductions in adiposity

Visceral obesity is considered to be harmful to health as compared with subcutaneous fat, it is most commonly associated with cardiovascular morbidity and mortality^10^. Compared with baseline, MES + HS reproduced specific reductions in visceral adiposity in obese type 2 diabetes as observed in the results of a preliminary crossover intervention^6^.

HSP72 induction increases lipolysis rates and activates oxidative enzymes, such as citrate synthase (CS) and β-hydroxyacyl CoA dehydrogenase (HAD) in liver as well as muscle^11^. Hyperthermia at 42 °C increases mitochondrial oxidative capacity with increased CS activity and cytochrome c oxidase activity in rat skeletal muscle^12^. It is also reported that HSP72 induction increases AMPK activation^6^. As AMPK knockdown increased fat mass and exacerbates the inflammatory phenotype^13^, activation of AMPK by MES + HS may reduce visceral adiposity. Indeed, lipid accumulation in *C. elegans* was attenuated by MES alone through liver kinase B1-AMPK signaling activation^14^. These lines of evidence suggest that HSP72 stimulates fat oxidation, resulting in reduced fat storage and adiposity. Indeed, whole body knockout of HSP72 mice exhibit obesity, insulin resistance, and marked lipid accumulation in skeletal muscle^15^.

The reason for preferential decreases in visceral fat versus subcutaneous fat by MES + HS treatment may be explained by the nature of those fat tissues. Using lifestyle modification interventions, obese men have significantly greater reductions in visceral fat mass^16^. Aerobic exercise has been shown to reduce visceral adipose mass in obesity, independent of weight loss. As activation of HSR by MES + HS stimulates similar pathways on exercise—such as activation of AMPK, sirt1, and peroxisome proliferator activated receptor γ coactivator-1α^17^ —this may explain the similar results of MES + HS treatment in preferential reductions in visceral fat.

### Greater improvements in men: sex differences

In the present study, improvements in metabolic parameters and body composition were mostly greater in male than in female participants. Although most male participants in this study presented a slight VFA dominant obesity (VFA/SFA = 50.1%), female patients exhibited far more SFA dominant (VFA/SFA = 29.8%). The distribution pattern of the accumulated fat may explain the sex differences. Another possibility would be a sex difference in basal HSP72 expression, which is higher in women than in men^18^. Hence, the activation of HSR may not fully influence the metabolic advantages in women. In addition, heat treatment activates androgen effects especially in lipid peroxidation in men^6^. These hypotheses of sex differences have to be elucidated in the near future. Of note, the reduction in HbA1c in male participants was 0.44% (*n* = 42) in the present study. Our previous report showed comparable decreases of HbA1c (0.43%) in male participants (*n* = 40)^6^, suggesting that MES + HS treatment reproduced reliable effects in glucose control in male subjects with type 2 diabetes.

### Amelioration of glucose control, inflammation, and insulin resistance

The major reason for the amelioration of glucose homeostasis using this intervention is the attenuation of insulin resistance, which is tightly associated with chronic systemic inflammation. Obese type 2 diabetes patients develop visceral obesity with insulin resistance mainly because of inflammatory cytokine production by activation of c-jun N-terminal kinase (JNK) and nuclear factor-kappa B (NF-κB). These molecules interfere with insulin signaling, resulting in inactivating downstream targets such as glucose transporter (GLUT) 4 and glycogen synthase kinase (GSK) 3β. GSK3β is a key molecule to activate heat shock factor (HSF)-1, which is a transcription factor for HSP72. HSP72 is a significant inhibitor of cytokine production by attenuating stress molecule JNK via multiple aspects^19^, such as direct association between HSP72 and JNK^20^, blocking upstream kinase signal-regulated kinase 1^20^, suppression of mitogen activated protein kinase (MAPK) kinase 1/7^21^, and enhancement of ubiquitin ligase C-terminus of Hsc70 interacting protein mediated dual leucine zipper-bearing kinase 1 ubiquitination^22^ and activation of MAPK phosphatase 1/3^23^.

Another important inflammatory activator is NF-κB, which is a transcription factor for CRP and TNF-α. We observed decreased expression and fewer nuclear localization of NF-κB in monocytes extracted from MES + HS treated type 2 diabetes patients compared with those before the treatment^6^. The decreased expression of NF-κB may be the result of reduced TNF-α expression because these two molecules positively regulate each other. Moreover, NF-κB nuclear translocation by metabolic stress is regulated by activation of IκB-kinase (IKK) and phosphorylation of NF-κ light polypeptide gene enhancer in B-cells inhibitor α(IκBα), which are negatively regulated by HSP72^24^. When MES + HS treatment is performed, cytoplasmic HSP72 levels are increased and act as an inhibitor of JNK and NF-κB signals, resulting in the attenuation of cytokine production, thereby improving insulin resistance. In addition, activation of AMPK by MES^14^ may directly activate GLUT4 translocation independent of insulin signaling. To note, the reduction in visceral adiposity is not correlated with the decrease in HbA1c, glucose levels or HOMA-IR, indicating that MES + HS treatment may directly attenuate the inflammatory signaling cascade to improve glucose homeostasis.

### Improvement in blood pressure, renal function, hepatic steatosis, and lipid profile

Systolic blood pressure showed a small but significant decrease in MES + HS treatment. This observation is supported that heat shock has been observed to improve blood pressure in primates^25^. Glucose normalization, as well as attenuation of inflammation, is an important intervention for diabetic nephropathy. We found that MES + HS treatment has a protective role for renal function in a mouse model of Alport syndrome by attenuation of inflammatory cytokine expression^26^. In a diabetic mouse model and in humans, we observed significant reductions in inflammatory signaling and urinary albumin excretion by MES + HS treatment^6^,^7^. These results indicate that MES + HS intervention has renal protective effects in addition to glucose control. MES + HS therapy also improves surrogate markers of hepatic steatosis and lipid profiles in this study. HSP72 may function to activate fatty acid oxidation as a result of activation of CS and β-HAD in liver and muscle, thereby reducing hepatic steatosis. AMPK activation and mitochondrial activation may also contribute to improved hepatic lipid accumulation. Additional effects of MES + HS with current DPP-4 inhibitor treatment are observed in FPG, L-FABP, and hepatic steatosis. DPP-4 inhibitors exert anti-inflammatory effect and anti-accumulation of hepatic lipids in mice^27^, suggesting that MES + HS treatment may enhance the benefits of DPP-4 inhibitors.

The limitation of this study is relatively small sample size and no setting of appropriate placebo control because this apparatus simultaneously delivers heat and mild electric stimulation, both of which are easily recognized by subjects. To overcome these problems, we are planning to design a larger clinical trail containing appropriate placebo setting of this MES + HS. Despite the lack of placebo control, two times treatment frequency could be used for the adjustment of the results in seven times per week of MES + HS treatment. As in Table 3, HbA1c was significantly decreased when adjusted by two times per week group (−0.55%. p = 0.001), indicating MES + HS did indeed improve glucose control in obese type 2 diabetes.

In summary, this research provides additional evidence to support the positive impacts of MES + HS treatment in improving metabolic outcomes in obese patients with type 2 diabetes. As most of these patients were already treated with anti-diabetic medications including DPP-4 inhibitors, patients who did not reach the glycemic control goal of HbA1c 7.0% could be offered additional personalized medical care including MES + HS treatment.

## Methods

### Study participants

A total of 63 obese Japanese patients with type 2 diabetes (43 men and 20 women) were recruited at six Japanese hospitals (online only material). Obesity with type 2 diabetes was defined as a waist circumference (Wc) >85 cm in men and >90 cm in women, with HbA1c levels from 7.0% to 9.3%. Participants ranged in age from 40 to 74 years and had been receiving stable medication(s) for at least 3 months. Exclusion criteria were shown in online only material (Supplementaly Table 1).

The study protocol complied with the ethical guidelines of the Declaration of Helsinki, and written informed consent was obtained from each subject. This study was approved by the institutional review board at Kumamoto University (Ethics No. 794). The trial was registered with an approved ICMJE clinical trial registry, UMIN (ID: UMIN 000016309) and the date of registration is Jan.23/2015.

### MES + HS treatment

The devices (BioMetronome^®^) for producing MES + HS were provided by Tsuchiya Rubber Co. Ltd. (Kumamoto, Japan). The description of the MES + HS device has been provided previously^6^. Briefly, MES + HS produces electrical stimulation of 1.4 ± 0.1 V/cm. The pads were positioned on the front and back of the abdomen and delivered 55 pulses per second, 0.1 msec duration with 42 °C heat. The padded area was 15 cm in length × 25 cm in width. MES + HS treatment was taken place at each subject’s home, and complete adherence were confirmed by logs that the subjects were instructed to fill out when they treated.

### Randomization and masking

Sixty-two eligible type 2 diabetes subjects were randomly assigned using computer-generated random numbers into three groups by block randomization. Finally, a total 60 subjects completed the study and were analyzed. All subjects or investigators were masked to treatment allocation at the time of enrollment.

### Study design and clinical protocol

This study was a prospective, randomized, open-label, triple-arm trial. The final 60 participants were randomly assigned into 12-week intervention period of MES + HS twice (*n* = 22), four times (*n* = 19) or seven times (*n* = 19) per week for 60 min per single treatment. Exercise and diet alterations were prohibited during the entire period. At 0 and 12 weeks, body compositions, abdominal adiposity, and metabolic and biochemical examinations were investigated.

The primary endpoints were the amount of visceral adiposity measured as described below, glucose control assessed by HbA1c, and insulin resistance estimated by HOMA-IR. Other outcomes include blood pressure, systemic inflammation, renal function, hepatic steatosis, and lipids. For the primary outcome, we estimated the need to enroll 54 subjects to detect changes in the visceral fat area of 15% with MES + HS as compared with no treatment, with statistical power of 80%, allowing for a type I (α) error of 0.05. Allowing for a loss to follow-up rate of 10%, 60 subjects were required to undergo randomization.

### Monocytes isolation and analysis

To investigate the HSP72 expression in monocyte from T2DM subjects, 6 subjects were randomly selected. Before and after 12 weeks of MET treatment (seven times per week group), blood samples were collected during fasted state. First, peripheral blood mononuclear cells (PBMCs) were isolated using BD Vacutainer^TM^ CPT^TM^ (BD, Franklin Lakes, NJ). Monocytes were subsequently isolated from the PBMCs magnetically by depletion technique (Miltenyl Biotech. Auburn. CA). Western blot was performed to confirm the expression levels of HSP72 as described in elsewhere^6^.

### Statistical analysis

Statistical analysis was performed with SPSS software (IBM, Chicago, IL, USA). All values were expressed as means ± standard error of the mean. The treatment effects of MES + HS were analyzed using a paired *t*-test or a Wilcoxon signed-rank test. Multiple comparisons were performed using one-way ANOVA. Two-sided *p*-values of less than 0.05 were considered to indicate statistical significance.

## Additional Information

**How to cite this article**: Kondo, T. *et al*. Activation of heat shock response to treat obese subjects with type 2 diabetes: a prospective, frequency-escalating, randomized, open-label, triple-arm trial. *Sci. Rep.* **6**, 35690; doi: 10.1038/srep35690 (2016).

## Supplementary Material

Supplementary Information

## Figures and Tables

**Table 1:** Baseline characteristics of participants.

	2 per week		4 per week		7 per week	
The number of participants	22		19		19	
Age (years)	57·9 ± 9·3		60·7 ± 9·9		59·3 ± 10·0	
Female (%)	36·4		26·3		26·3	
Diabetes history (years)	7·6 ± 5·2		8·4 ± 5·5		9·0 ± 4·3	
Medications	ratio (%)		ratio (%)		ratio (%)	
SU	6	27·3	10	52·6	11	57·9
BG	10	45·5	11	57·9	15	78·9
α-GI	1	4·5	4	21·1	3	15·8
glinide	2	9·1	1	5·3	1	5·3
TZD	1	4·5	2	10·5	5	26·3
DPP-4 inhibitor	12	54·5	13	68·4	13	68·4
SGLT2 inhibitor	0	0·0	0	0·0	0	0·0
Adiposity						
Visceral Fat Area (cm^2^)	151·05 ± 11·84		170·85 ± 9·14		178·85 ± 17·45	
SubQ Fat Area (cm^2^)	207·3 ± 17·89		206·00 ± 23·52		207·99 ± 21·01	
total Fat Area (cm^2^)	358·35 ± 25·82		376·85 ± 25·99		386·83 ± 33·34	
BMI (kg/m^2^)	28·54 ± 0·93		29·22 ± 0·83		29·74 ± 1·10	
Wc (cm)	99·03 ± 1·99		98·90 ± 1·91		102·55 ± 2·87	
Glucose control and insulin resistance						
Fasting plasma glucose (mg/dL)	150·73 ± 8·77		169·95 ± 9·70		158·84 ± 8·50	
Fasting IRI (µIU/mL)	9·28 ± 1·12		10·94 ± 1·41		9·16 ± 1·32	
HOMA-IR	3·58 ± 0·62		4·79 ± 0·77		3·95 ± 0·81	
HbA1c (%)	7·44 ± 0·13		7·74 ± 0·13		7·77 ± 0·15	
GA (%)	17·99 ± 0·53		19·42 ± 0·67		20·22 ± 0·86	
Adiponectin (µg/mL)	7·05 ± 0·76		6·88 ± 0·72		8·76 ± 0·90	
Blood pressure						
Systolic Blood Pressure (mmHg)	131·77 ± 3·27		133·89 ± 3·45		138·05 ± 2·52	
Diastolic Blood Pressure (mmHg)	77·05 ± 2·19		78·58 ± 1·88		79·74 ± 2·45	
Heart Rate (bpm)	76·59 ± 2·71		78·47 ± 2·24		75·53 ± 3·60	
Systemic inflammation						
TNF-α (pg/mL)	1·53 ± 0·16		1·65 ± 0·14		1·77 ± 0·24	
IL-6 (pg/mL)	3·02 ± 0·56		3·11 ± 0·48		3·41 ± 0·42	
hs-CRP (ng/mL)	1793·23 ± 590·35		3070·74 ± 1151·81		1070·05 ± 232·37	
WBC (/µL)	6900·00 ± 412·61		6747·37 ± 320·52		6584·21 ± 291·07	
Renal function						
eGFR (mL/min/1.73 m^2^)	76·07 ± 3·22		76·53 ± 4·77		77·03 ± 3·57	
ACR (µg/gCre)	79·11 ± 48·76		63·55 ± 23·05		82·32 ± 24·82	
L-FABP (mg/gCr)	4·52 ± 0·93		5·47 ± 0·85		6·41 ± 0·90	
Hepatic steatosis and lipids						
AST/ALT	0·77 ± 0·05		0·83 ± 0·06		0·97 ± 0·06	
UA (mg/dL)	5·64 ± 0·37		5·82 ± 0·31		5·28 ± 0·28	
LDL-C (mg/dL)	108·36 ± 6·18		113·63 ± 7·077		106·05 ± 4·70	
HDL-C (mg/dL)	50·55 ± 3·04		53·00 ± 3·31		54·21 ± 3·14	
TG (mg/dL)	185·95 ± 29·78		192·63 ± 36·04		171·21 ± 31·71	
FFA (µEq/L)	617·64 ± 40·25		570·42 ± 49·68		721·63 ± 64·72	

**Table 2:** Effects of MES + HS over time compared with baseline.

Adiposity				
	Baseline	MES + HS	delta	p value
Visceral Fat Area (cm^2^)	166.12 ± 7.75	154.44 ± 6.76	−11.69	**
SubQ Fat Area (cm^2^)	207.11 ± 11.95	204.04 ± 12.14	−3.07	0.205
total Fat Area (cm^2^)	373.23 ± 16.47	358.48 ± 16.07	−14.75	**
BMI (kg/m^2^)	29.14 ± 0.56	28.88 ± 0.58	−0.25	**
Wc (cm)	100.10 ± 1.33	97.86 ± 1.23	−2.24	**
Glucose control and insulin resistance				
Fasting plasma glucose (mg/dL)	159.38 ± 5.30	144.45 ± 4.21	−14.93	**
Fasting IRI (µIU/mL)	9.77 ± 0.74	7.91 ± 0.59	−1.86	**
HOMA-IR	4.08 ± 0.42	2.99 ± 0.29	−1.09	**
HbA1c (%)	7.64 ± 0.08	7.28 ± 0.08	−0.36	**
GA (%)	19.15 ± 0.41	18.40 ± 0.39	−0.75	**
Adiponectin (µg/mL)	7.54 ± 0.47	8.47 ± 0.54	0.93	**
Blood pressure				
Systolic Blood Pressure (mmHg)	134.43 ± 1.84	132.08 ± 1.83	−2.35	*
Diastolic Blood Pressure (mmHg)	78.38 ± 1.27	77.10 ± 1.40	−1.28	0.139
Heart Rate (bpm)	76.85 ± 1.68	75.02 ± 1.44	−1.83	0.052
Systemic inflammation				
TNF-α (pg/mL)	1.64 ± 0.11	1.24 ± 0.08	−0.40	**
IL-6 (pg/mL)	3.17 ± 0.29	2.78 ± 0.31	−0.44	0.105
hs-CRP (ng/mL)	1968.77 ± 442.90	1305.17 ± 246.04	−663.60	**
WBC (/µL)	6751.67 ± 204.86	6315.00 ± 183.01	−436.67	**
Renal function				
eGFR (mL/min/1.73 m^2^)	76.52 ± 2.23	79.48 ± 2.35	2.96	**
ACR (µg/gCre)	75.20 ± 20.87	56.47 ± 15.82	−18.73	*
L-FABP (mg/gCr)	5.42 ± 0.53	4.38 ± 0.49	−1.04	**
Hepatic steatosis and lipids				
AST/ALT	0.86 ± 0.03	0.92 ± 0.04	0.06	**
UA (mg/dL)	5.58 ± 0.19	5.43 ± 0.18	−0.15	*
LDL-C (mg/dL)	109.30 ± 3.54	105.80 ± 3.45	−3.50	0.055
HDL-C (mg/dL)	52.48 ± 1.84	52.92 ± 1.65	0.43	0.354
TG (mg/dL)	183.40 ± 18.75	153.38 ± 12.46	−30.02	*
FFA (µEq/L)	635.62 ± 30.80	584.02 ± 25.73	−51.60	0.064

p value; *<0.05, **<0.01.

**Table 3:** Effects of MES + HS depending on frequency of treatment.

Adiposity	2 per week			4 per week			7 per week			P values		
	Baseline	MES + HS	delta	Baseline	MES + HS	delta	Baseline	MES + HS	delta	2 vs 4	4 vs 7	2 vs 7
Visceral Fat Area (cm^2^)	151.05 ± 11.84	145.69 ± 11.08	−5.37	170.85 ± 9.14	156.62 ± 9.01	−14.24	178.85 ± 17.45	162.39 ± 14.20	−16.45	0.071	0.389	0.054
SubQ Fat Area (cm^2^)	207.30 ± 17.89	201.47 ± 17.04	−5.83	206.00 ± 23.52	203.45 ± 23.67	−2.55	207.99 ± 21.01	207.61 ± 22.79	−0.38	0.287	0.422	0.300
total Fat Area (cm^2^)	358.35 ± 25.82	347.16 ± 25.09	−11.19	376.85 ± 25.99	360.07 ± 26.64	−16.79	386.83 ± 33.34	370.00 ± 31.74	−16.84	0.258	0.498	0.341
BMI (kg/m^2^)	28.54 ± 0.93	28.29 ± 0.96	−0.25	29.23 ± 0.83	29.04 ± 0.87	−0.18	29.74 ± 1.10	29.42 ± 1.15	−0.33	0.338	0.242	0.350
Wc (cm)	99.03 ± 1.99	97.45 ± 1.84	−1.57	98.90 ± 1.91	96.53 ± 1.94	−2.37	102.55 ± 2.87	99.66 ± 2.55	−2.89	0.242	0.329	0.100
Glucose control and insulin resistance												
Fasting plasma glucose (mg/dL)	150.73 ± 8.77	142.41 ± 7.87	−8.32	169.95 ± 9.70	152.89 ± 7.75	−17.05	158.84 ± 8.50	138.37 ± 5.29	−20.47	0.116	0.356	0.093
Fasting IRI (µIU/mL)	9.28 ± 1.12	8.30 ± 1.11	−0.98	10.94 ± 1.41	8.65 ± 0.92	−2.30	9.16 ± 1.32	6.73 ± 0.91	−−2.43	0.133	0.460	0.137
HOMA-IR	3.58 ± 0.62	3.16 ± 0.55	−0.42	4.79 ± 0.77	3.40 ± 0.51	−1.39	3.95 ± 0.81	2.39 ± 0.41	−1.56	*	0.415	0.067
HbA1c (%)	7.44 ± 0.13	7.34 ± 0.15	−0.10	7.74 ± 0.13	7.37 ± 0.15	−0.36	7.77 ± 0.15	7.12 ± 0.10	−0.65	0.058	*	**
GA (%)	17.99 ± 0.53	17.66 ± 0.40	−0.33	19.42 ± 0.67	18.77 ± 0.77	−0.64	20.22 ± 0.86	18.87 ± 0.83	−1.34	0.297	0.151	0.085
Adiponectin (µg/mL)	7.05 ± 0.76	7.69 ± 0.79	0.63	6.88 ± 0.72	7.72 ± 0.78	0.83	8.76 ± 0.90	10.13 ± 1.10	1.37	0.328	0.169	0.114
Blood pressure												
Systolic Blood Pressure (mmHg)	131.77 ± 3.27	130.09 ± 3.17	−1.68	133.89 ± 3.45	132.95 ± 3.53	−0.95	138.05 ± 2.52	133.53 ± 2.67	−4.53	0.430	0.204	0.234
Diastolic Blood Pressure (mmHg)	77.05 ± 2.19	77.86 ± 2.66	0.82	78.58 ± 1.88	78.42 ± 1.97	−0.16	79.74 ± 2.45	74.89 ± 2.38	−4.84	0.358	0.050	*
Heart Rate (bpm)	76.59 ± 2.71	73.82 ± 1.79	2.77	78.47 ± 2.24	76.42 ± 1.93	−2.05	75.53 ± 3.60	75.00 ± 3.53	−0.53	0.398	0.258	0.233
Systemic inflammation												
TNF-α (pg/mL)	1.53 ± 0.16	1.21 ± 0.12	−0.32	1.65 ± 0.14	1.22 ± 0.11	−0.43	1.77 ± 0.24	1.31 ± 0.17	−0.46	0.320	0.431	0.269
IL-6 (pg/mL)	3.02 ± 0.56	2.69 ± 0.61	−0.33	3.11 ± 0.48	2.46 ± 0.34	−0.78	3.41 ± 0.42	3.18 ± 0.57	−0.23	0.230	0.228	0.448
hs-CRP (ng/mL)	1793.23 ± 590.35	1434.00 ± 488.96	−359.23	3070.74 ± 1151.81	1645.53 ± 494.28	−1425.21	1070.05 ± 232.37	815.63 ± 138.15	−254.42	0.086	0.338	0.076
WBC (/µL)	6900.00 ± 412.61	6563.64 ± 308.12	−336.36	6747.37 ± 320.52	6310.53 ± 270.52	−436.84	6584.21 ± 291.07	6031.58 ± 354.32	−552.63	0.370	0.321	0.245
Renal function												
eGFR (mL/min/1.73 m^2^)	76.07 ± 3.22	78.35 ± 3.49	2.28	76.53 ± 4.77	77.98 ± 4.78	1.45	77.03 ± 3.57	81.03 ± 3.95	4.00	0.307	0.086	0.137
ACR (mg/gCre)	79.11 ± 48.76	64.48 ± 37.31	−14.63	63.55 ± 23.05	58.32 ± 23.11	−5.23	82.32 ± 24.82	45.35 ± 9.20	−36.97	0.247	0.181	0.083
L-FABP (µg/gCr)	4.52 ± 0.93	3.86 ± 0.66	−0.66	5.47 ± 0.85	5.21 ± 1.22	−0.26	6.41 ± 0.90	4.15 ± 0.52	−2.25	0.315	0.055	*
Hepatic steatosis and lipids												
AST/ALT	0.77 ± 0.05	0.83 ± 0.05	0.06	0.83 ± 0.06	0.93 ± 0.09	0.10	0.97 ± 0.06	1.00 ± 0.07	0.02	0.257	0.160	0.247
UA (mg/dL)	5.64 ± 0.37	5.40 ± 0.37	−0.24	5.82 ± 0.31	5.59 ± 0.29	−0.23	5.28 ± 0.28	5.32 ± 0.23	0.04	0.479	0.097	0.103
LDL-C (mg/dL)	108.36 ± 6.18	109.68 ± 5.86	1.32	113.63 ± 7.07	110.00 ± 6.81	−3.63	106.05 ± 4.70	97.11 ± 4.55	−8.95	0.191	0.135	*
HDL-C (mg/dL)	50.55 ± 3.04	50.82 ± 2.34	0.27	53.00 ± 3.31	53.84 ± 3.54	0.84	54.21 ± 3.14	54.42 ± 2.61	0.21	0.416	0.427	0.490
TG (mg/dL)	185.95 ± 29.78	155.23 ± 25.40	−30.73	192.63 ± 36.04	162.63 ± 16.09	−30.00	171.21 ± 31.71	142.00 ± 20.32	−29.21	0.492	0.492	0.473
FFA (µEq/L)	617.64 ± 40.25	592.45 ± 42.66	−25.18	570.42 ± 49.68	576.84 ± 41.58	6.42	721.63 ± 64.72	581.42 ± 49.25	−140.21	0.324	0.099	*

p value; *<0.05, **<0.01.

**Table 4:** Effects of MES + HS in sex differences.

					vs. Baseline	F vs. M
Adiposity	Group	Baseline	MES + HS	delta	p-value	p-value
Visceral Fat Area (cm^2^)	F	140.10 ± 6.51	137.44 ± 7.49	−2.66	0.262	*
	M	177.23 ± 10.25	161.72 ± 8.87	−15.55	**	
SubQ Fat Area (cm^2^)	F	276.76 ± 21.76	266.17 ± 21.30	−10.59	0.068	0.092
	M	177.25 ± 11.56	177.41 ± 12.70	0.16	0.485	
total Fat Area (cm^2^)	F	416.86 ± 26.77	403.61 ± 25.28	−13.25	0.050	0.425
	M	354.53 ± 19.85	339.14 ± 19.50	−15.39	0.012	
BMI (kg/m^2^)	F	30.07 ± 1.09	29.92 ± 1.16	−0.15	0.068	0.197
	M	28.74 ± 0.64	28.44 ± 0.65	−0.30	**	
Wc (cm)	F	100.16 ± 2.04	98.92 ± 1.95	−1.24	0.117	0.071
	M	100.08 ± 1.69	97.40 ± 1.54	−2.67	**	
Glucose control and insulin resistance						
Fasting plasma glucose (mg/dL)	F	140.44 ± 5.25	142.44 ± 6.77	2.00	0.313	**
	M	167.50 ± 6.86	145.31 ± 5.27	−22.19	**	
Fasting IRI (µIU/mL)	F	10.47 ± 1.36	8.51 ± 1.08	−1.96	0.032	0.450
	M	9.47 ± 0.88	7.65 ± 0.70	−1.82	**	
HOMA-IR	F	3.72 ± 0.54	3.16 ± 0.48	−0.57	0.077	0.123
	M	4.23 ± 0.56	2.92 ± 0.37	−1.31	**	
HbA1c (%)	F	7.50 ± 0.13	7.33 ± 0.15	−0.17	0.140	0.033
	M	7.70 ± 0.10	7.25 ± 0.10	−0.44	**	
GA (%)	F	17.77 ± 0.66	17.89 ± 0.76	0.12	0.384	*
	M	19.74 ± 0.49	18.61 ± 0.46	−1.12	**	
Adiponectin (µg/mL)	F	8.09 ± 0.80	9.16 ± 0.75	1.07	*	0.341
	M	7.30 ± 0.58	8.17 ± 0.69	0.87	**	
Blood pressure						
Systolic Blood Pressure (mmHg)	F	131.89 ± 3.73	134.28 ± 2.76	2.39	0.229	*
	M	135.52 ± 2.06	131.14 ± 2.32	−4.38	*	
Diastolic Blood Pressure (mmHg)	F	77.78 ± 2.41	79.89 ± 2.27	2.11	0.244	*
	M	78.64 ± 1.50	75.90 ± 1.71	−2.74	**	
Heart Rate (bpm)	F	78.44 ± 3.37	77.72 ± 3.25	−0.72	0.366	0.258
	M	76.17 ± 1.90	73.86 ± 1.48	−2.31	*	
Systemic inflammation						
TNF-α (pg/mL)	F	1.53 ± 0.18	1.18 ± 0.12	−0.36	*	0.372
	M	1.69 ± 0.13	1.27 ± 0.10	−0.42	**	
IL-6 (pg/mL)	F	3.13 ± 0.64	3.69 ± 0.78	0.56	0.209	**
	M	3.19 ± 0.31	2.38 ± 0.26	−0.87	**	
hs-CRP (ng/mL)	F	1985.83 ± 609.06	1890.78 ± 553.03	−95.06	0.287	0.084
	M	1961.45 ± 576.37	1054.19 ± 249.73	−907.26	**	
WBC (/µL)	F	6883.33 ± 366.94	6544.44 ± 245.73	−338.89	0.070	0.293
	M	6695.24 ± 246.31	6216.67 ± 237.69	−478.57	**	
Renal function						
eGFR (mL/min/1.73 m^2^)	F	81.07 ± 3.06	83.40 ± 3.29	2.33	*	0.413
	M	74.57 ± 2.84	77.23 ± 3.00	2.66	**	
ACR (µg/gCre)	F	90.81 ± 30.13	65.43 ± 20.73	−25.38	0.132	0.304
	M	68.51 ± 26.81	52.63 ± 20.75	−15.88	*	
L-FABP (mg/gCr)	F	5.26 ± 0.90	4.28 ± 0.92	−0.97	*	0.460
	M	5.49 ± 0.65	4.42 ± 0.58	−1.06	*	
Hepatic steatosis and lipids						
AST/ALT	F	0.84 ± 0.04	0.89 ± 0.05	0.04	0.094	0.339
	M	0.86 ± 0.04	0.93 ± 0.05	0.07	*	
UA (mg/dL)	F	4.87 ± 0.25	4.80 ± 0.23	−0.07	0.318	0.278
	M	5.89 ± 0.24	5.70 ± 0.22	−0.18	*	
LDL-C (mg/dL)	F	119.17 ± 7.41	115.72 ± 6.55	−3.44	0.171	0.493
	M	105.07 ± 3.75	101.55 ± 3.88	−3.52	0.101	
HDL-C (mg/dL)	F	60.28 ± 2.85	56.72 ± 2.21	−3.56	0.087	*
	M	49.14 ± 2.12	51.29 ± 2.11	2.14	*	
TG (mg/dL)	F	185.83 ± 38.13	137.28 ± 11.67	−48.56	0.094	0.186
	M	182.36 ± 21.22	160.29 ± 16.97	−22.07	*	
FFA (µEq/L)	F	693.39 ± 56.83	685.67 ± 42.19	−7.72	0.455	0.198
	M	610.86 ± 35.98	540.45 ± 29.56	−70.40	*	

p value; *<0.05, **<0.01.
