# Supplementary material for: Activation of heat shock response to treat obese subjects with type 2 diabetes: a prospective, frequency-escalating, randomized, open-label, triple-arm trial
Source: Sci Rep. 2016 Oct 19;6:35690. doi: 10.1038/srep35690 (PMC5069544; doi:10.1038/srep35690)
Supplement: Supplementary Information [file srep35690-s1.pdf]

Activation of heat shock response to treat obese subjects with type 2 diabetes: a prospective, frequency-escalating, randomized, open-label, triple-arm trial

Tatsuya Kondo[1], Rieko Goto[1], Kaoru Ono[1], Sayaka Kitano[1], Mary Ann Suico[2], Miki Sato[1], Motoyuki Igata[1], Junji Kawashima[1], Hiroyuki Motoshima[1], Takeshi Matsumura[1], Hirofumi Kai[2], Eiichi Araki[1]*.

Supplementary Information

Contents

- **Listing of Study Participating Investigators**
- **Supplementary Table 1. Complete Inclusion/Exclusion criteria**
- **Supplementary Table 2. Flow Diagram of Subjects Disposition**
- **Supplementary Table 3. The results of MES+HS with or without DPP-4 inhibitors.**

- **Listing of Study Participating Investigators**

This investigation was a multicenter study participating of 6 hospitals. The name of the institute and participating investigators are shown below.

1. Kumamoto University Hospital. E Araki, T. Kondo, N. Furukawa, S. Shimoda, T. Matsumura, H. Motoshima, J. Kawashima, D. Kukidome, M. Igata, N. Ishii, S. Iwashita, Y. Nishioka, R Goto, R. Matsuyama, K. Ono, S. Kitano.
2. Arao Municipal Hospital. K Sasaki.
3. Tamana Central Hospital. N. Miyamura, H. Matsuda.
4. Kikuchi Medical Association Hospital. T. Yano, T. Taguchi.
5. Kumamoto General Hospital. T. Sekigami.
6. Minamata City General Hospital and Medical Center. R. Tanaka.

- **Supplementary Table 1. Complete Inclusion/Exclusion criteria**

Inclusion criteria

1. Male and female subjects who are ≧ 40 and < 75 years old.
2. Waist circumference: ≧ 85 cm in male and ≧ 90 cm in female.
3. HbA1c: ≧ 7.0% and < 9.4%
4. Oral anti diabetic medications do not matter.

Exclusion criteria

1. Type 1 diabetes.
2. Secondary forms of diabetes.
3. Acute or severe chronic diabetic complications.
4. Insulin therapy or other injectable medical treatment.
5. Past history or current medication of myocardial infarction, angina, apoplexy and arteriosclerosis obliterans.
6. Severe wound, infections, pre- or post-operation.
7. Severe liver dysfunction (AST and/or ALT: greater than 100U).
8. Chronic heart failure, familial dyslipidemia.
9. Pacemaker carrier.

- **Supplementary Table 2. Flow Diagram of Subjects Disposition**

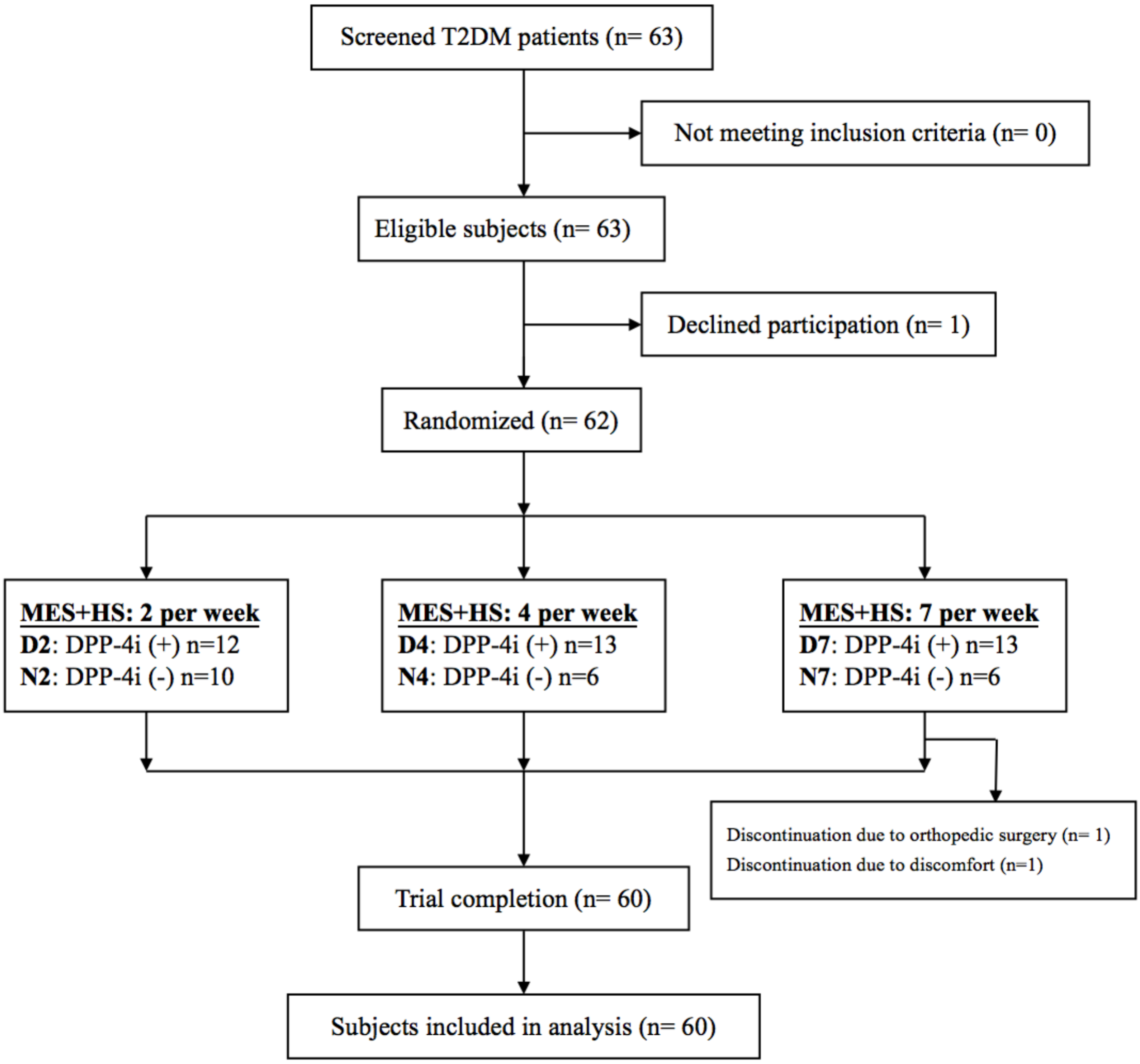

One potential male participant declined to continue after the acquisition of informed consent.

One participant (female, designated into the seven treatments per week group) discontinued treatment owing to orthopedics surgery independent of this intervention and another (male, designated into the seven treatments per week group) discontinued treatment because of discomfort from electrical stimulation.

- **Supplementary Table 3. The results of MES+HS with or without DPP-4 inhibitors.**

**Adiposity**									**vs. Baseline**	**D vs. N**
	**Group**	**Baseline**			**MES+HS**			**delta**	**p-value**	**p-value**
Visceral Fat Area (cm^2)	D	169.01	±	8.99	158.39	±	7.70	-10.62	**	0.311
	N	161.15	±	14.29	147.62	±	12.63	-13.53	**	
SubQ Fat Area (cm^2)	D	213.66	±	14.41	209.70	±	14.86	-3.97	0.234	0.376
	N	195.78	±	20.81	194.28	±	20.75	-1.51	0.351	
total Fat Area (cm^2)	D	382.67	±	19.60	368.08	±	19.46	-14.59	*	0.483
	N	356.93	±	29.19	341.90	±	27.78	-15.04	*	
BMI (kg/m^2)	D	29.76	±	0.62	29.54	±	0.63	-0.23	*	0.334
	N	28.06	±	1.05	27.76	±	1.11	-0.30	*	
Wc (cm)	D	100.40	±	1.63	97.94	±	1.44	-2.46	**	0.263
	N	99.59	±	2.28	97.72	±	2.24	-1.87	**	
**Glucose control and insulin resistance**										
	**Group**	**Baseline**			**MES+HS**			**delta**	**p-value**	**p-value**
Fasting plasma glucose (mg/dL)	D	162.16	±	6.96	142.29	±	5.41	-19.87	**	*
	N	154.59	±	7.92	148.18	±	6.62	-6.41	0.082	
Fasting IRI (μIU/mL)	D	10.07	±	0.80	7.67	±	0.63	-2.40	**	0.085
	N	9.26	±	1.47	8.33	±	1.18	-0.93	0.162	
HOMA-IR	D	4.26	±	0.49	2.89	±	0.34	-1.36	**	0.108
	N	3.78	±	0.78	3.16	±	0.54	-0.61	0.107	
HbA1c (%)	D	7.50	±	0.09	7.09	±	0.09	-0.41	**	0.157
	N	7.87	±	0.14	7.60	±	0.14	-0.27	*	
GA (%)	D	18.90	±	0.56	17.86	±	0.48	-1.04	**	0.082
	N	19.57	±	0.57	19.32	±	0.64	-0.25	0.335	
Adiponectin (μg/mL)	D	7.86	±	0.61	8.78	±	0.71	0.93	**	0.495
	N	6.99	±	0.74	7.92	±	0.78	0.93	**	
**Blood pressure**										
	**Group**	**Baseline**			**MES+HS**			**delta**	**p-value**	**p-value**
Systolic Blood Pressure (mmHg)	D	135.45	±	2.44	132.29	±	2.34	-3.16	0.080	0.263
	N	132.68	±	2.68	131.73	±	2.93	-0.95	0.351	
Diastolic Blood Pressure (mmHg)	D	78.89	±	1.56	77.97	±	1.79	-0.92	0.251	0.344
	N	77.50	±	2.16	75.60	±	2.18	-1.91	0.199	
Heart Rate (bpm)	D	77.82	±	2.35	76.05	±	2.03	-1.76	0.140	0.467
	N	75.18	±	2.06	73.23	±	1.72	-1.95	0.066	
**Systemic inflammation**										
	**Group**	**Baseline**			**MES+HS**			**delta**	**p-value**	**p-value**
TNF−α (pg/mL)	D	1.63	±	0.13	1.29	±	0.11	-0.33	**	0.166
	N	1.67	±	0.18	1.16	±	0.09	-0.51	**	
IL-6 (pg/mL)	D	3.21	±	0.40	2.80	±	0.39	-0.41	0.072	0.437
	N	3.10	±	0.37	2.73	±	0.53	-0.50	0.370	
hs-CRP (ng/mL)	D	2078.71	±	610.42	1389.55	±	361.06	-689.16	*	0.451
	N	1778.86	±	587.20	1159.41	±	244.53	-619.45	0.114	
WBC (/μL)	D	6847.37	±	275.44	6352.63	±	257.11	-494.74	**	0.257
	N	6586.36	±	289.58	6250.00	±	227.16	-336.36	0.052	
**Renal function**										
	**Group**	**Baseline**			**MES+HS**			**delta**	**p-value**	**p-value**
eGFR (mL/min/1.73m^2)	D	73.87	±	2.88	76.25	±	3.02	2.38	**	0.363
	N	81.10	±	3.27	83.98	±	3.48	2.88	*	
ACR (mg/gCre)	D	87.60	±	30.60	68.07	±	24.24	-19.53	*	0.451
	N	53.77	±	20.35	10.76	±	1.86	-17.35	0.175	
L-FABP (μg/gCr)	D	5.65	±	0.76	4.09	±	0.62	-1.56	**	*
	N	5.02	±	0.58	4.88	±	0.80	-0.14	0.418	
**Hepatic steatosis and lipids**										
	**Group**	**Baseline**			**MES+HS**			**delta**	**p-value**	**p-value**
AST/ALT	D	0.85	±	0.04	0.94	±	0.06	0.09	**	*
	N	0.86	±	0.05	0.87	±	0.05	0.01	0.451	
UA (mg/dL)	D	5.82	±	0.25	5.58	±	0.23	-0.24	*	0.085
	N	5.18	±	0.27	5.18	±	0.27	0.01	0.486	
LDL-C (mg/dL)	D	106.32	±	4.11	102.61	±	4.06	-3.71	0.107	0.450
	N	114.45	±	6.38	111.32	±	6.11	-3.14	0.162	
HDL-C (mg/dL)	D	52.26	±	2.07	52.66	±	1.98	0.39	0.368	0.483
	N	52.86	±	3.52	53.36	±	2.92	0.50	0.420	
TG (mg/dL)	D	179.26	±	24.38	154.58	±	16.37	-24.68	0.109	0.303
	N	190.55	±	28.94	151.32	±	18.83	-39.23	**	
FFA (μEq/L)	D	643.84	±	38.04	569.82	±	32.53	-74.03	*	0.192
	N	621.41	±	52.22	608.55	±	41.53	-12.86	0.422	

D: DPP-4 inhibitor (+), N: DPP-4 inhibitor (-)

p value; *: <0.05, **: <0.01
